# Evaluation of the impact of breastfeeding support groups in primary health CENTRES in Andalusia, Spain: a study protocol for a cluster randomized controlled trial (GALMA project)

**DOI:** 10.1186/s12889-020-09244-w

**Published:** 2020-07-18

**Authors:** Rodríguez-Gallego Isabel, Leon-Larios Fatima, Ruiz-Ferron Cecilia, Lomas-Campos Maria-de-las-Mercedes

**Affiliations:** 1 Virgen del Rocío University Hospital (Seville), Centro Universitario de Enfermería Cruz Roja, University of Seville, Sevilla, Spain; 2 Nursing Department, Faculty of Nursing, Physiotherapy and Podiatry, University of Seville, Sevilla, Spain

**Keywords:** Breastfeeding, Social network, Lactation support, Support group

## Abstract

**Background:**

In 2003, the World Health Organization recommended exclusive breastfeeding (EB) during the newborn’s first 6 months of life and, if possible, during the first 2 years. However, EB rates resist these recommendations. In developed countries, only 1 out of 3 babies is breastfed during its first 6 months of life, and great differences between areas and countries can be observed. Only 35% of the newborns receive breastfeeding at 3–4 months of age. There are diverse strategies described in the literature that have proven their efficiency in improving breastfeeding rates. It has also been proven that professional support is an effective tool to extend any kind of breastfeeding; besides, it has been observed that mother-to-mother support also increases breastfeeding initiation, sustainment, and exclusive duration. The overall aim of the study is to assess the impact of the support groups on the sustainment of exclusive breastfeeding until 6 months after birth.

**Methods/design:**

This study is a cluster-random multicentric clinical trial with a control group and an intervention group, without blinding because it is impossible to mask the intervention. A randomization by centres of primary health (clusters) will be carried out. The women allocated to the intervention or control group will be randomized with a simple randomization sampling. The participants’ breastfeeding rate will be followed up at the first 10 days, and at 2, 4, and 6 months of their newborn’s life.

**Discussion:**

There is a need to assess the impact of mother support groups on exclusive breastfeeding. This study aims to analyse the outcomes related to the support received and to identify what should the structure of these groups be; in other words, to describe factors related to a better breastfeeding experience in order to help women increase breastfeeding rates.

**Trial registration:**

The trial is prospectively recorded at the ISRCTN registry (Trial ID: ISRCTN17263529). Date recorded: 17/06/2020.

## Background

In 2003, the World Health Organization (WHO) recommended exclusive breastfeeding (EB) during the newborn’s first 6 months of life and, if possible, during the first 2 years [1]. Breastfeeding is considered the best option to feed newborns due to its multiple properties. However, EB rates resist these recommendations. In developed countries, only 1 out of 3 babies is breastfed during its first 6 months of life, and great differences between areas and countries can be observed [2]. Only 35% of the newborns receive breastfeeding at 3–4 months of age [3].

The percentage of newborns that initiate breastfeeding in their first hour of life has significant importance, since it has been proven that early initiation of breastfeeding after birth is associated with a longer duration of this practice [4]. Despite all the benefits that have been described in the previous literature, for the mother as well as for the newborn at a physical and psychosocial level [5–8], breastfeeding rates after hospital discharge are roughly 71% [9], with a considerable decrease in the first weeks of the newborn’s life, reaching 25% at 6 months postpartum [10]. However, some studies suggest that these women felt disappointed because they could not prolong breastfeeding any longer. Usually, women who ceased breastfeeding after the first 6 weeks postpartum would have wanted to continue for longer, as well as those who breastfed for at least 6 months [1]. The main reasons described were lack of knowledge and of milk production [2]. Thus, it is brought to light that women have a positive inclination to continue breastfeeding but, due to diverse reasons, they are forced to give it up.

A recently published systematic review (2015) identifies the barriers and facilitators related to breastfeeding sustainment; experiences with previous births, certain birth-related hospital practices, or the fact that the mother had been breastfed by her own mother are some of the factors that condition the current breastfeeding experience [11].

Furthermore, there are also factors such as maternal age, schooling level, family support, multiparity or antenatal lessons, that can condition prolongation [12]. On the other hand, it has been observed how other collectives are more vulnerable to breastfeeding sustainment, such as those who have a low socioeconomic status or immigrants, who require greater support from the health professionals [13].

Some studies have been carried out assessing the strategies that can increase mother adherence to breastfeeding, and a positive effect was observed. Professional support has been proven to be effective in order to extend any kind of breastfeeding, but the effects on EB are less clear [11, 14]. Besides, it has been observed that mother-to-mother support for breastfeeding also increases breastfeeding initiation, sustainment, and exclusive duration [15].

The types of support described in the studies are diverse: from educational interventions to advice during home visits, support among breastfeeding mothers, telephone support, and educational programs on the Internet, as well as breastfeeding advice during the mother’s stay at the hospital [2, 16]. All these studies have proven to be effective in some way, but it has also been concluded that we need more studies assessing the effectiveness of professional and non-professional support in different contexts, particularly in communities with low rates of initiation and sustainment of breastfeeding, and in women who desire to continue for more than 3 months [17].

For this reason, we outline this study, which aims to assess the impact that joining breastfeeding support groups has on breastfeeding sustainment and prolongation in primary health centres of the Spanish Autonomous Community of Andalusia.

## Methods/design

The overall aim of the study is to assess the impact of the support groups on the sustainment of exclusive breastfeeding until the first 6 months after birth. More specifically we will:

- Determine factors related to breastfeeding sustainment.
- Identify the motivators of breastfeeding interruption in infants during the first 6 months of life.
- Describe the main problems consulted by women in the breastfeeding support groups.
- Compare the strategies of face-to-face and virtual ante/postnatal sessions with only postnatal sessions regarding breastfeeding sustainment.
- Know the influence of these support groups on the perceived self-efficiency of breastfeeding.

### Study design

This is a multicentric cluster randomized controlled trial with a control group and an intervention group, without blinding. This protocol has been written according to the recommendations of the SPIRIT 2013 statement [18, 19], a guideline that defines the standard elements of a protocol, including recommendations for intervention trials, which can be found in Additional file 1. Besides, the design of this clinical trial follows the requirements of the CONSORT statement.

#### Participants, recruitment, and study area

Eligible female participants will be recruited in randomized primary health centres (clusters) in Andalusia, Spain. Andalusia is an Autonomous Community divided into 8 provinces with a total of 8,414,240 inhabitants (available data in 2019) [20], with a birth rate of 8.48 for each 1000 inhabitants (2018) [21]. By July 1st, 2019, the number of females in a reproductive age in Andalusia was 1,924,680 [22]. This is a multicentric study based on the Andalusian provinces of Seville, Huelva, Cádiz, Granada, and Jaén. Participant recruitment will be conducted on the antenatal care clinic at the 35th–37th week of pregnancy. Midwives will explore the women’s intentions regarding breastfeeding and whether or not they meet the inclusion criteria described below; they will also explain to them the purpose of the study including its risks and benefits. The women will be invited to participate voluntarily.

The eligibility criteria will be revised in the first postpartum visit for the women who accept to participate in both the control and the experimental groups.

Midwives meeting the criteria described below will serve as trained recruiters:

- At least 2 years of professional experience as a midwife
- Not having the intention of abandoning the work centre during the project time period
- Attendance to the project’s training meeting
- Signature of the research agreement

#### Inclusion criteria

1. Healthy women with exclusive or partial breastfeeding 10 days after birth, and who attended antenatal lessons in the Primary Health Centre
2. Women over 18 years of age
3. Women who accepted and signed the informed consent

#### Exclusion criteria

1. HIV positive
2. Cancer
3. Tuberculosis infection
4. No intention to breastfeed
5. Impossibility or contraindication to breastfeed due to medical conditions
6. Communication difficulties due to language

#### Randomization and data management

Centres are randomized to either an intervention arm or normal care. After this randomization, women are allocated either to the intervention group or to the control group by simple random sampling at the 35th–37th week of pregnancy by the midwives at the primary health centres, who will confirm that these women have the intention to breastfeed and that they have indeed initiated it in the first 10 days postpartum. Once the centre randomization for the recruitment of women for the control and intervention groups is conducted, these will be once again randomized following a simple randomization strategy. The participants’ recruitment will begin in September 2020, and will end in March 2022. This will be carried out by those midwives responsible for each health centre, being advised by one of the project’s experts.

These experts will work independently of the accountable researchers for the recruitment of participants. They will allocate health centres using a random sequence made by the *Oxford Minimization and Randomization (OxMAR)* system [23]. The experts will randomly assign a non-transferrable identifier to the health centres, making a difference between the control group and the experimental group centres. Subsequently, each participant will have an identification code depending on their group. This way, we intend to avoid the bias between two women attending antenatal care at the same centre who may or may not join the breastfeeding support group linked thereto, depending on whether they are allocated to one group or the other.

### Description of the intervention

#### Intervention group

The women in the intervention group will have the usual antenatal care, this is, at least one group lesson with theoretical-practical content about breastfeeding, as a part of the antenatal education taught by their reference health centre’s midwife, trained for that purpose.

Subsequently, in the first ten days after birth, they will have a preliminary individual appointment with their midwife, where individual doubts will be answered and obstetrical-neonatal data, related to birth and this subsequent period, will be collected. Data referred to in the WHO’s BREASTfeeding Observation Form [24], in the General Self-Efficacy Scale [25], and in the Breastfeeding Self-Efficacy Scale [26] will be collected as well.

The women will participate in monthly face-to-face, two-hour group sessions, called Breastfeeding Support Groups, where the midwife will act as a leading figure and moderator. These sessions will have an educational component, using theoretical-practical demonstrations related to breastfeeding themes, based on the Baby-Friendly Hospital Initiative (BFHI) [27] recommendations**.** Besides, there will be a motivational element and a component based on the social or the mother-to-mother support established in the group. During these sessions, the women will be able to share their concerns, experiences, and doubts and, furthermore, they will help each other with their own knowledge about breastfeeding. Should they find any difficulties, the midwife will take part as an expert on the topic, and as a moderator when answers are given among mothers with successful breastfeeding experiences. Thus, they will be offered monthly face-to-face, professional-led, organized, and proactive support.

Apart from these monthly meetings, the participants will have the choice of interacting with each other, with other lactating women, and with the reference midwife, using Facebook and WhatsApp groups created for that purpose. This way, mother-to-mother support can be reinforced and the resolution of doubts on the subject through Information and Communication Technologies (ICTs) [28] can be assessed. They will also have the option of arranging an individual appointment with their reference midwife, as well as the usual postnatal care. All of this will be recorded.

A follow-up at the first two, four, and six months after birth will be performed; at these points, the main outcome variables will be collected (current way of feeding, dropout register, obstacles, ICTs use register, neonatal admission, return to work) and the same scales as in the first visit, previously mentioned, will be once again administered.

#### Control group

The women in the control group will receive the usual antenatal care according to the pregnancy, birth, and postpartum care protocol of the Department of Equality, Health and Social Policies of the Regional Government of Andalusia [29], regarding antenatal and postnatal care, as well as the women in the intervention group.

Besides, they will have the option of asking for individual on-demand appointments with their reference midwife at the health centre they belong to.

The first postpartum visit will take place before the first ten days and the follow-up will be performed after the first two, four, and six months after birth; at these points, the main outcome variables will be collected (current way of feeding, dropout register, obstacles, ICTs use register, neonatal admission, return to work) and the same scales as in the first visit, previously mentioned in the intervention group, will be once again administered [24–26].

**Fig. 1:**
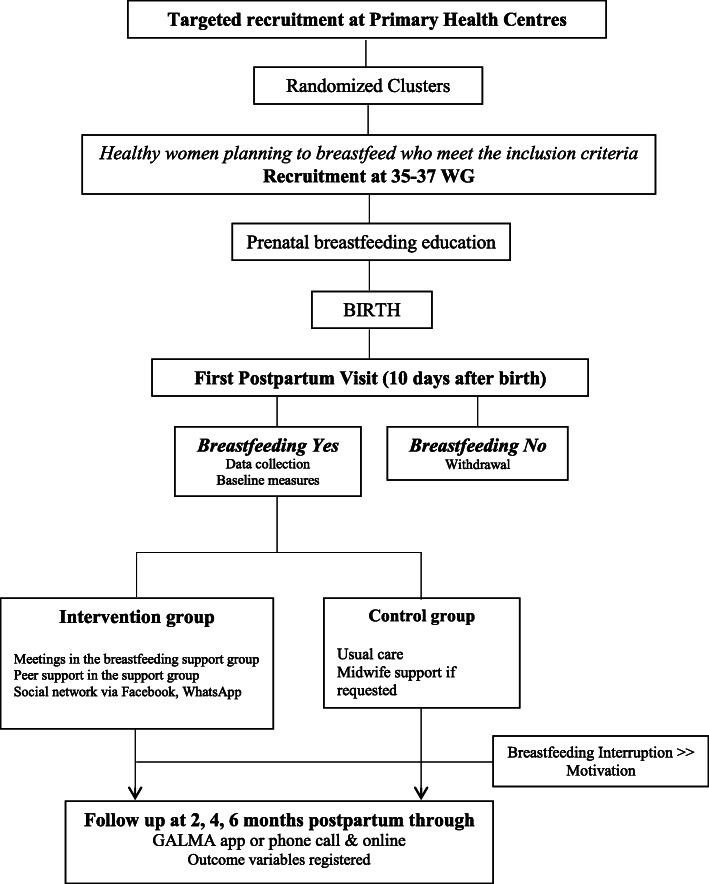
Flow diagram of the participants through the trial

Figure 1 details the flow of participants from recruitment until the last follow-up contact for control and intervention subjects.

#### Data collection

Once the woman is randomized to the control or intervention group and has signed the written consent, having been previously informed of the study objectives, the midwife will let her know about the follow-up she will need to perform throughout the study. The woman will have to choose whether she prefers to be followed up via the GALMA Project application (App), or by filling in online questionnaires via WhatsApp®.

The previously mentioned basal follow-up will be conducted and the participants will have to facilitate the information using the study App, which will have previously sent a reminder message to them. Those women who opt to fill in online questionnaires will receive a phone call the previous day from a member of the research team, who will send to them the link to the questionnaires. Data related to the monitoring, both electronic and via the App, will be coded and saved by the research team. Those data related to obstetrical results and those derived from a hospital stay will be drawn from the patient’s medical record, after the patient’s consent.

Incorrect or missing data will be checked by the members of the research team and the participants will be asked for their fulfilment. All data will be recorded in an electronic database with access only by the team members. Hard copies of the participants’ questionnaires will be kept in a safe and secure file which can be accessed only with previous authorization.

### Outcome measurements

#### Primary outcomes measurement

Type of infant feeding after the first ten days, 2, 4, and 6 months of life, depending on whether they are in the control or in the intervention group.

- Exclusive breastfeeding: it means that the newborn is fed only with breast milk, without using any other milk or food, from its birth up until the first 6 months of its corrected age, carrying out the measuring at those intervals corresponding to 2, 4, and 6 months. Breastfeeding duration is defined as the period that goes from birth until the last administration of breast milk [8].
- Nearly exclusive breastfeeding: occasional administration of formula milk [8].
- Partial breastfeeding: combination of breast and formula milk [8].
- Formula feeding: exclusively formula milk [8].

#### Secondary outcomes measurement

Differences between both groups with respect to the following:

- ***Sociodemographic outcomes:*** mother’s age, schooling level (without any studies, primary, secondary, University studies), marital status (single, married, divorced, widow), work status (self-employed, employed, unemployed), occupation, working hours/week, home country.
- ***Breastfeeding outcomes:***
  - *Breastfeeding observation form (BREAST):* it is a tool developed by a WHO experts committee in 1992 [24], which collects the key points in order to assess a breastfeed through observation: body position (B), responses (R), emotional bonding (E), anatomy (A), suckling (S), and time spent suckling (T). In the Baby-Friendly Hospital Initiative (BFHI) strategy [27], it is specified that a complete breastfeed observation by an expert is essential for checking whether there is an appropriate transfer and for diagnosing the cause of possible difficulties, should there be any, for further resolution.
  - *Breastfeeding Self-Efficacy:* a reduced version of the *Breastfeeding Self-Efficacy Scale-Short Form* (BSES-SF) [30] scale [26] which was validated in Spanish by Oliver-Roi et al. [26], and counts on 14 items grouped in only one dimension. It is a one-dimensional, self-completed scale of 14 items, positively presented and preceded by the sentence ‘I always can’. For scoring, it has a Likert-type scale from 1 to 5, where 1 indicates ‘not sure at all’ and 5 indicates ‘very sure’. Higher scores mean higher self-efficacy levels for breastfeeding. The BSES-SF Spanish version’s reliability, measured with the Cronbach alpha coefficient, is 0.92. It is a valid and reliable measure, successfully adapted into diverse cultural contexts, including the Spanish [26, 31].
  - *Previous breastfeeding experiences*: multiparous women will be asked about their experience with breastfeeding while raising previous children, as well as the reason for giving it up.
  - *Type of infant feeding after hospital discharge*: exclusive breastfeeding, nearly exclusive breastfeeding, partial breastfeeding, formula feeding.
  - *Common breastfeeding problems and solutions* (consultation with the midwife, Internet, social networks, reference person …).
  - *Reasons for breastfeeding interruption:* mother’s decision, low weight gain, mother’s disease, newborn’s disease, contraindication due to medication, nipple problems, difficulties with the technique, return to work, etc.
- ***Obstetric outcomes:*** mode of birth (eutocic/dystocic/C-section), mode of start of labour (spontaneous/induced), analgesia during labour (epidural/others), perineal trauma classification (episiotomy/perineal tear degree), initiation of skin-to-skin contact immediately after birth.
- ***Neonatal outcomes:***
  - *Perinatal aspects*: newborn’s gender (female/male), weight (expressed in grams), Apgar test score [32], admission in neonatal care unit (yes/no).
  - *Newborn’s health problems in the first 6 months of life:* description for subsequent coding.
- ***Behaviour outcomes:***
  - *General Self-Efficacy*: this scale assesses optimistic self-beliefs of coping efficiently with a variety of difficult demands in life. Baessler and Schwarzer’s adaptation [33] will be used, although, in spite of its original format, a ten point Likert-type answer scale suggested by Sanjuan et al. [25] will be used, consisting of 10 items and a scoring range from 10 to 100, with an internal consistency of 0.87 (Alpha coefficient) and a correlation between two halves of 0.88.
- ***Outcomes related to the use of social networks for support:*** frequency of use of social networks and/or WhatsApp, number of times the resource is used, questions resolved through face-to-face appointment in the midwife clinic, use of the App for consultation regarding breastfeeding.

Table 1 shows the measurement tools used for data collection, the method of collection and time points.

**Table 1:** Measurement domains, survey tools, and collection time point

Variable	Component	Measurement tools/ Questions	Baseline	2mth	4mth	6mth
*Sociodemographic measures*		Age, schooling level, marital status, employment, ethnicity.	✓			
*Clinical information*	Obstetric outcomes	Type of birth, onset of labour, type of analgesia, perineal trauma, skin-to-skin technique	✓			
	Neonatal outcomes	Gender, weight, Apgar, neonatology admission, health problem	✓			
*Breastfeeding measures*	Previous experience	In affirmative case, interruption motivation	✓			
	Type of breastfeeding	Exclusive breastfeeding, nearly exclusive breastfeeding, partial breastfeeding, formula	✓	✓	✓	✓
	Breastfeeding information	Breastfeeding observation form (BREAST)	✓			
	Breastfeeding Self-Efficacy	Breastfeeding Self-Efficacy Scale-Short Form (BSES-SF)	✓	✓	✓	✓
	Breastfeeding problems	Type of problem and resolution (midwife, peer, social network, Internet …)	✓	✓	✓	✓
	Breastfeeding Interruption - Motivation			✓	✓	✓
*Behaviour measures*	Self-Efficacy General Scale		✓	✓	✓	✓
*Social networks (intervention group)*	Use and frequency			✓	✓	✓
	Problem consulted			✓	✓	✓
*Clinical services*	Appointments related to breastfeeding attended			✓	✓	✓

### Process evaluation

The follow-up will be conducted by a qualified researcher, independent from the study group, without any conflict of interest. They will verify the notification of participation in the study and the appropriate tracking of participants by the researchers.

During the whole study process, a continuous follow-up will be performed, which will allow for the identification of potential problems and solutions, implementing the required changes in a short term. This study will count on an expert researcher, responsible for monitoring the participant centres, as well as the participants’ compliance with the follow-up protocols. A global report about the study status will be elaborated biweekly with the lead researcher. The midwives recruiting patients in the health centres will be contacted monthly, in order to let them know the number of women recruited, as well as the follow-up situation performed. All the study data will be included in an electronic database by the research team. All information will be pre-coded and kept safe in a database. During the research, the lead researcher, along with the expert, will have access to that database.

### Withdrawal

Any participant can withdraw from the study at any moment and by any given reasons, either because she repeatedly breaks the follow-up protocol or, if she is allocated to the intervention group, because she does not want to keep on attending the breastfeeding support group sessions any longer. Anyway, the withdrawal reason will be kept for the record for further study. The participants who withdraw from the study will not be replaced.

### Study sample size

Taking into account that the EB rate in Andalusia is 25%, this is considered the expected value in the control group. An estimated increase effect of 12% of the EB rates at 6 months is established, as asserted in previous studies [34]. To reach this difference between both groups, a two-tail hypothesis is set out, with a power of 80% and assuming a type I error of 5%, assuming a loss of 10% in the follow-up, the necessary sample increases to 512 women, 256 in each group. Missing values in the follow-up will be addressed by multiple imputation technique, performing attrition analysis.

### Adverse events

Adverse events are currently not foreseen, due to the study and intervention’s nature.

### Analysis

A complete statistical analysis plan will be previously performed, as well as a report thereof and of the results, according to the Consolidated Standards of Reporting Trials (CONSORT) [35, 36]. The analysis will be carried out on the basis of an intention-to-treat principle, without taking into account whether or not adherence to the breastfeeding support group was completed. Individual health centres are the randomization units and the mother-newborn dyad will be the analysis unit. All statistical trials and confidence intervals will be assumed with a type I error established in alpha = 0.05, using the IBM SPSS V22 statistics package [37].

An exploratory data analysis will be conducted in order to identify atypical values, for the total of our studied sample, as well as for the different sub-groups created. A graphic exploratory analysis of the different studied variables will also be conducted, according to their numerical or non-numerical character. Frequency and percentage distribution scales will be elaborated for the descriptive analysis of variables with a qualitative (non-numerical) character. For the quantitative (numerical) ones, centralization and dispersion measures will be calculated. In variables with a normal distribution, the mean and the standard deviation will be calculated and, in those with an asymmetric distribution, the mean and interquartile range (p25-p75) will be calculated. Furthermore, point estimators and confidence intervals will be calculated at 95% for averages and percentages. The relationship between two qualitative variables will be analysed with Pearson’s chi-square test, the correction for continuity of the chi-square test, a linear-by-linear association test, and Fisher’s exact probability test, based on the application criteria. The significant results of these tests will be complemented with a confidence interval set at 95% for percentage differences.

Regarding the numerical variables between two groups, the Student’s t-test of independent samples for mean comparisons will be carried out, once the requirements of randomness, normality (Kolmogorov-Smirnov’s test, or Shapiro-Wilks’ test, according to the sample size) and equality of variances (Levene’s test) are validated. In case this one is not achieved, the Student’s t-test for independent samples with Welch’s correction will be carried out. In case normality requirements are not accomplished, the Mann-Whitney’s U test will be applied.

Should any statistically significant differences be considered, confidence intervals at 95% for mean differences will be determined. For the comparison of numerical variables between more than two groups, and once the independence, normality, and homoscedasticity requirements are validated, the ANOVA (analysis of variance) test will be conducted; in case the requirements are not achieved, the Kruskal-Wallis’ test will be carried out. Should any statistically significant differences between the means of the different studied sub-groups be detected, multiple comparison tests will be conducted in order to identify between which sub-groups were those differences found, considering a corrected level of alpha significance based on the number of comparisons made. On the survival analysis, the Kaplan-Meier’s non-parametric estimator will be used.

The effectiveness analysis will be conducted through the comparison of the number of women who chose to breastfeed exclusively at 6 months in both groups, using Fisher’s exact test and estimating the confidence intervals of the difference. Should factors be unequal in both groups, an explanatory logistic regression model would be carried out, in which the dependent variable would be exclusive breastfeeding, and the independent one would be the intervention group, adjusted for the potential factor findings. The efficacy analysis would be carried out on an intention-to-treat basis.

In order to avoid the effect of the intervention on the duration of breastfeeding in the support group, a survival analysis will be conducted, which will compare the intervention and control groups using the log-rank test. Control of potential confounders will be made through Cox’s different regression models. All *p* values under 0.05 will be considered as statistically significant.

### Ethics and dissemination

This study has been elaborated in strict compliance with the ethical principles of the Declaration of Helsinki (1964), subsequently revised in Tokyo (1975), Venice (1983), Hong Kong (1989), Somerset West (1996), Edinburgh (2000), Washington (2002), Seoul (2008), and Fortaleza (2013), including the informed consent request for the women participating in it.

Participation in the project is voluntary, as well as the participation request. The study has been laid out according to Spanish regulation act No. 14/2007 of July 3rd, regarding biomedical research, complying with the protocol suitability requirements and with the procedure regarding the study objectives. In the same way, the study will be conducted according to the Declaration of Helsinki.

All patient-related data collected for this study will be treated according to the Spanish Organic Law on Protection of Personal Data (Spanish Organic Law 15/1999) of December 13th and to European Regulation (EU) 2016/679 of the European Parliament and Council of April 27th, 2016, as well as to the established confidentiality and anonymity rules.

A written informed consent was provided to every participant in the study. The women were informed of the study objectives before being allocated to an arm.

The study was approved by the Research Ethics Committees of the Virgen Macarena and Virgen del Rocío hospitals (Seville, Spain) on February 24th, 2020 (Code 1936-N^− 19^).

## Discussion

There is no breastfeeding follow-up nor tracking system in Spain. Data come from studies conducted by health professionals at the regional level and they do not allow for a correct assessment nor for following up in time. Spanish statistics suggest that the breastfeeding time average is 3–5 months, the biggest amount of withdrawals happening over the first 4 weeks, and the second biggest one between the third and the fourth month, due to the mother’s return to work [38].

Taking these limitations into account, and according to the data collected from the Spanish National Health Survey (*Encuesta Nacional de Salud*), the breastfeeding rate in Spain (including partial breastfeeding and EB) at the first 6 weeks has been kept mostly stable, with global numbers around 71%, while in the last 15 years it has been possible to observe a progressive increase of breastfeeding at 3 (66.5% in the year 2012) and 6 months of life (46.9%). On the other hand, the EB percentage at 6 months in 2012 was over 28.5%, a percentage similar to the European global numbers but far from the WHO-UNICEF recommendations [39].

International studies have proved that 54% of newborns had received formula milk at some time in their first week of life, and the vast majority of them, in their first year of life [40]. This brings to light how usual it is to complement breastfeeding with formula milk. In this sense, the improvement of the professionals’ training on breastfeeding and the promotion of family and social support for breastfeeding mothers are two key points in order to increase the prevalence of EB [41].

There already are breastfeeding support groups led by midwives in Spain nowadays [42], but they do not follow a common structure or systematization that allows for impact assessment. This project aims to develop a common structure based on prenatal counselling, antenatal classes and a follow-up over the first 6 months of the newborn’s life, by means of monthly group sessions and mother-to-mother support through social networks (Facebook and WhatsApp), a support resource described in the previous literature [28], and to assess their efficacy. This will help elaborate a guide which can offer a direction to those professionals who wish to set up a breastfeeding support group in their primary health centre.

Imdad et al. [43] already suggested in their systematic review that support groups had a bigger impact on breastfeeding rates than individual counselling, and that results were better on every care level. On the other hand, it has been observed that prenatal counselling had a bigger impact, achieving better breastfeeding rates at 4–6 weeks postpartum, whereas the combination of prenatal and postnatal counselling extended breastfeeding duration up to the first 6 months, hence prenatal as well as postnatal training and counselling are advised in order to reach better rates.

It has been proven in other studies [40, 44, 45] how one-to-one peer support, when provided by a professional, has a bigger effect on the initiation, sustainment, and duration of breastfeeding, which reinforces the idea of breastfeeding mother groups led by midwives.

### Trial status

Recruitment will begin in September 2020.

## Supplementary information

**Additional file 1: Appendix A.** Spirit Statement Checklist.

## Data Availability

The datasets used and/or analysed during the current study are available from the corresponding author upon reasonable request.

## Abbreviations

APP: Application
BFHI: Baby-Friendly Hospital Initiative
BSES-SF: Breastfeeding Self-Efficacy Scale-Short Form
CONSORT: Consolidated Standards of Reporting Trials
GALMA: *Grupo de Apoyo a la Lactancia Materna Andalucía*
HIV: Human Immunodeficiency Virus
IBM-SPSS: International Business Machines-Statistical Package for Social Sciences
ICTs: Information and Communication Technologies
EB: Exclusive Breastfeeding
OxMAR: Oxford Minimization and Randomization
SPIRIT: Standard Protocol Items Recommendations for Interventional Trials
UNICEF: United Nations Children’s Fund
WHO: World Health Organization
