# Correction to: Evaluation of the impact of breastfeeding support groups in primary health centres in Andalusia, Spain: a study protocol for a cluster randomized controlled trial (GALMA project)

**DOI:** 10.1186/s12889-020-09528-1

**Published:** 2020-09-23

**Authors:** Isabel Rodríguez-Gallego, Fatima Leon-Larios, Cecilia Ruiz-Ferron, Maria-de-las-Mercedes Lomas-Campos

**Affiliations:** 1 Virgen del Rocío University Hospital (Seville), Centro Universitario de Enfermería Cruz Roja, University of Seville, Sevilla, Spain; 2 Nursing Department, Faculty of Nursing, Physiotherapy and Podiatry, University of Seville, Sevilla, Spain

**Correction to: BMC Public Health 20, 1129 (2020)**

**https://doi.org/10.1186/s12889-020-09244-w**

It was highlighted that in the original article [1] the given names and the family names of the authors were interchanged. The original article has been updated.
